# Comparison of Red-Complex Bacteria Between Saliva and Subgingival Plaque of Periodontitis Patients: A Systematic Review and Meta-Analysis

**DOI:** 10.3389/fcimb.2021.727732

**Published:** 2021-10-08

**Authors:** Yaling Jiang, Bingqing Song, Bernd W. Brandt, Lei Cheng, Xuedong Zhou, Rob A. M. Exterkate, Wim Crielaard, Dong Mei Deng

**Affiliations:** ^1^ State Key Laboratory of Oral Diseases & National Clinical Research Center for Oral Diseases & Department of Cariology and Endodontics, West China Hospital of Stomatology, Sichuan University, Chengdu, China; ^2^ Department of Preventive Dentistry, Academic Center for Dentistry Amsterdam (ACTA), University of Amsterdam and Vrije Universiteit Amsterdam, Amsterdam, Netherlands

**Keywords:** periodontitis, *Porphyromonas gingivalis*, *Tannerella forsythia*, *Treponema denticola*, 16S rRNA gene amplicon sequencing, real-time PCR

## Abstract

The development of periodontitis is associated with an imbalanced subgingival microbial community enriched with species such as the traditionally classified red-complex bacteria (*Porphyromonas gingivalis*, *Tannerella forsythia*, and *Treponema denticola*). Saliva has been suggested as an alternative to subgingival plaque for the microbial analysis due to its easy and non-invasive collection. This systematic review aims to determine whether the levels of red-complex bacteria assessed using saliva reflect those in subgingival plaque from periodontitis patients. The MEDLINE, EMBASE, and Cochrane Library databases were searched up to April 30, 2021. Studies were considered eligible if microbial data of at least one of the red-complex species were reported in both saliva and subgingival plaque from periodontitis patients, based on DNA-based methods. Of the 17 included studies, 4 studies used 16S rRNA gene sequencing techniques, and the rest used PCR-based approaches. The detection frequency of each red-complex species in periodontitis patients was reported to be > 60% in most studies, irrespective of samples types. Meta-analyses revealed that both detection frequencies and relative abundances of red-complex bacteria in saliva were significantly lower than those in subgingival plaque. Moreover, the relative abundances of all 3 bacterial species in saliva showed significantly positive correlation with those in subgingival plaque. In conclusion, current evidence suggests that one-time saliva sampling cannot replace subgingival plaque for microbial analysis of the red-complex bacteria in periodontitis patients. Given the positive microbial associations between saliva and subgingival plaque, a thorough review of longitudinal clinical studies is needed to further assess the role of saliva.

## 1 Introduction

Periodontitis is one of the most prevalent oral infectious diseases, affecting over 740 million people worldwide (Kassebaum et al., 2014). It is a chronic inflammation, associated with dysbiotic subgingival biofilms, resulting in progressive and irreversible destruction of tooth supporting tissues (Vieira Colombo et al., 2016; Tonetti et al., 2017). Although it is not clear whether dysbiotic biofilms initiate the disease or are a consequence of the disease, it is well established that at diseased status, the subgingival microbiota is enriched with gram-negative, proteolytic bacteria, while in healthy situation, the microbiota is mainly composed of gram-positive bacteria (Curtis et al., 2020; Van Dyke et al., 2020). A recent review collected existing evidence and proposed an “Inflammation-Mediated Polymicrobial-Emergence and Dysbiotic-Exacerbation” (IMPEDE) model, which included the microbial element and complemented the current clinical classification of periodontitis (Van Dyke et al., 2020). According to this model, local inflammation drives an initial shift in microbial composition and the formation of periodontal pocket exacerbates this microbial shift by further enriching disease-associated species.

To determine the compositional shift of periodontitis-related microbiota, subgingival plaque obtained from diseased pockets has been considered as the most representative sample. However, collecting subgingival plaque is invasive and requires specialized training for proper sampling. Moreover, reports have shown that the quality and quantity of collected plaque samples may be greatly influenced by the collection methods (Renvert et al., 1992; van der Horst et al., 2013). Compared to subgingival plaque, saliva is much better accessible and can be collected noninvasively in larger quantity. Since the collection of saliva does not require special sampling tools, sample quality could be less influenced by sampling methods or operators. Saliva has been proposed as an alternative to subgingival plaque for studying the association between oral microbes and periodontal disease since 1998 (Umeda et al., 1998). It was hypothesized that microbes residing in a periodontal pocket could be spread, washed out or spilt over into saliva (Haririan et al., 2014; Li et al., 2015). Multiple clinical studies have compared the levels of important periodontal microbes using samples collected from subgingival pockets and saliva, but the results were inconsistent. For example, Umeda et al. (1998) reported that *Porphyromonas gingivalis* and *Treponema denticola* were detected more often in saliva than in subgingival plaque samples, whereas Nickles et al. (2017) reported the opposite. Moreover, the open-ended DNA sequencing techniques developed in the past decades have revealed that different niches in the oral cavity harbor considerably different microbial communities with distinct microbial composition (Huttenhower et al., 2012; Mark Welch et al., 2019). Differential microbial profiles of saliva and subgingival plaque have been demonstrated by several studies (Segata et al., 2012; Simón-Soro et al., 2013). Therefore, a systematic review that includes recent studies using sequencing techniques is needed to objectively assess microbial compositions of saliva and subgingival plaque.

So far, the most frequently examined microbes in clinical samples obtained from periodontitis patients are *P. gingivalis*, *Tannerella forsythia* and *T. denticola*. These 3 species were grouped as red-complex bacteria in 1998 (Socransky et al., 1998) based on the evidence that they were frequently isolated together and were strongly associated with periodontitis. Members of the red-complex group have been the main target in many clinical studies which performed the comparison between saliva and subgingival plaque samples. In the past, techniques employed to examine the levels of these bacteria were mainly targeted approaches, such as bacterial culture, immunological assays, PCR and quantitative real-time PCR (qPCR) (Suchett-Kaye et al., 2001). Among them, PCR and qPCR were used most frequently, since these techniques have better sensitivity and specificity as compared to other methods (Loesche, 1992; Boutaga et al., 2003). In addition, the abovementioned 16S rRNA gene sequencing techniques have been increasingly applied in clinical studies (Li et al., 2015; Belstrøm et al., 2017), since they offer the possibility to profile the entire microbiota besides specific bacterial species of interest.

This systematic review aimed to evaluate clinical evidence on the levels of red-complex bacteria in saliva and subgingival plaque samples collected from patients with periodontitis. We focused on studies which used DNA-based (targeted and open-ended) methods for microbial identification.

## 2 Materials and Methods

This systematic review was performed following the Preferred Reporting Items for Systematic Reviews and Meta-analysis (PRISMA) statement (Moher et al., 2009), and was registered at the National Institute for Health Research PROSPERO, International Prospective Register of Systematic Reviews (registration number: CRD42020219510).

### 2.1 Search Strategy

The MEDLINE (*via* PubMed), EMBASE, and Cochrane Library databases were searched up to April 30, 2021 by two independent researchers (YJ and BS), using the search strategy described in **Supplementary Table S1**. In addition, a manual search of the reference list of the included studies was conducted.

### 2.2 Study Selection

Studies that met the following criteria were included: 1) population: humans with periodontitis; 2) exposure: saliva samples; 3) comparison: subgingival plaque samples; 4) outcome: microbial data of at least one of the red-complex bacteria that was obtained using a DNA-based method; 5) study design: clinical studies of any design, except case report and case series.

Exclusion criteria were: 1) studies without periodontal diagnosis of the participants; 2) studies including participants who had an explicit diagnosis of any systemic disease or systemic condition, such as pregnancy; 3) studies including participants who used medication (e.g., antibiotics) or the medication status of participants was not mentioned; 4) studies with no baseline data in case of a prospective or interventional design; 5) not full-text publications (e.g., conference abstracts); 6) studies published in languages other than English. In addition, publications with overlapped data were identified at the phase of full-text screening. These studies were conducted by the same group of authors, and the data (a part or all) reported in different publications were obtained from the same group of subjects. In order to avoid duplicate data extraction, only the publication on a larger number of subjects was included.

The selection of studies was performed in two steps based on the above inclusion and exclusion criteria. In the first step, articles were screened on the basis of title and abstract, using a Web platform (rayyan.qcri.org) (Ouzzani et al., 2016). In the second step, the selected studies underwent full-text evaluation.

### 2.3 Data Extraction and Methodological Quality Assessment

A customized data extraction form was used to collect the following information from each included study: 1) characteristics of the study (e.g., author, year of publication, and study location); 2) characteristics of the participants (e.g., number, periodontal diagnosis, and clinical parameters); 3) methodological features of the study (e.g., method of saliva and subgingival plaque sample collection, method to evaluate red-complex bacteria); 4) microbial outcomes. For prospective and interventional studies, only data from the baseline measurement were extracted for analysis. When needed, the corresponding author(s) were contacted for the missing data.

The methodological qualities of all included studies were assessed using the 8-item “Critical Appraisal Checklist for Analytical Cross-Sectional Studies” by the Joanna Briggs Institute (JBI) (Moola et al., 2020). The answer to each question was “Yes”, “No”, or “Unclear”, and an overall rating score was given to each study which equals to the total number of “Yes” answers given, ranging from 0 to 8. The scores of 0–3, 4–5, and 6–8 were classified as low, medium, and high quality of studies, respectively (Yazdanian et al., 2020).

The steps mentioned above, including study selection, data extraction and methodological quality assessment were conducted independently by two researchers (YJ and BS), and any disagreement was resolved through discussion. If disagreement persisted, another researcher (DD) was consulted to achieve consensus.

### 2.4 Summary Outcome Measures and Statistical Analysis

Based on the results of included studies, we summarized 3 types of microbial outcomes: detection frequency, bacterial count and/or relative abundance of each red-complex bacteria.

Detection frequency refers to the percentage of subjects positive for a specific microorganism. It was reported in studies using either targeted PCR-based approaches or 16S rRNA gene sequencing techniques. Bacterial count provides the (semi-) quantity of a specific microorganism in a sample. This outcome parameter was reported in the studies using qPCR techniques. For studies using 16S rRNA gene sequencing techniques, two types of data were extracted: detection frequency and relative abundance.

Meta-analyses were performed to assess the statistical differences in the detection frequency and relative abundance of each red-complex species between saliva and subgingival plaque samples, using RevMan software version 5.4 (The Cochrane Collaboration; Copenhagen, Denmark). Heterogeneity among studies was assessed by chi-squared test and inconsistency index *I^2^*. Values of *I^2^* < 30%, 30%–60%, and > 60% were considered as low, moderate and large heterogeneity, respectively (Higgins et al., 2003). When the heterogeneity was significant (*p* < 0.1), a random-effects model (DerSimonian-Laird method) was applied to examine the overall effect; otherwise, a fixed-effects model (Mantel-Haenszel method) was used. The odds ratio (OR) for detection frequency and mean difference (MD) for relative abundance were calculated at 95% confidence intervals (CI). Differences were considered statistically significant if *p* < 0.05.

## 3 Results

### 3.1 Results of Search and Study Selection

**Figure 1:**
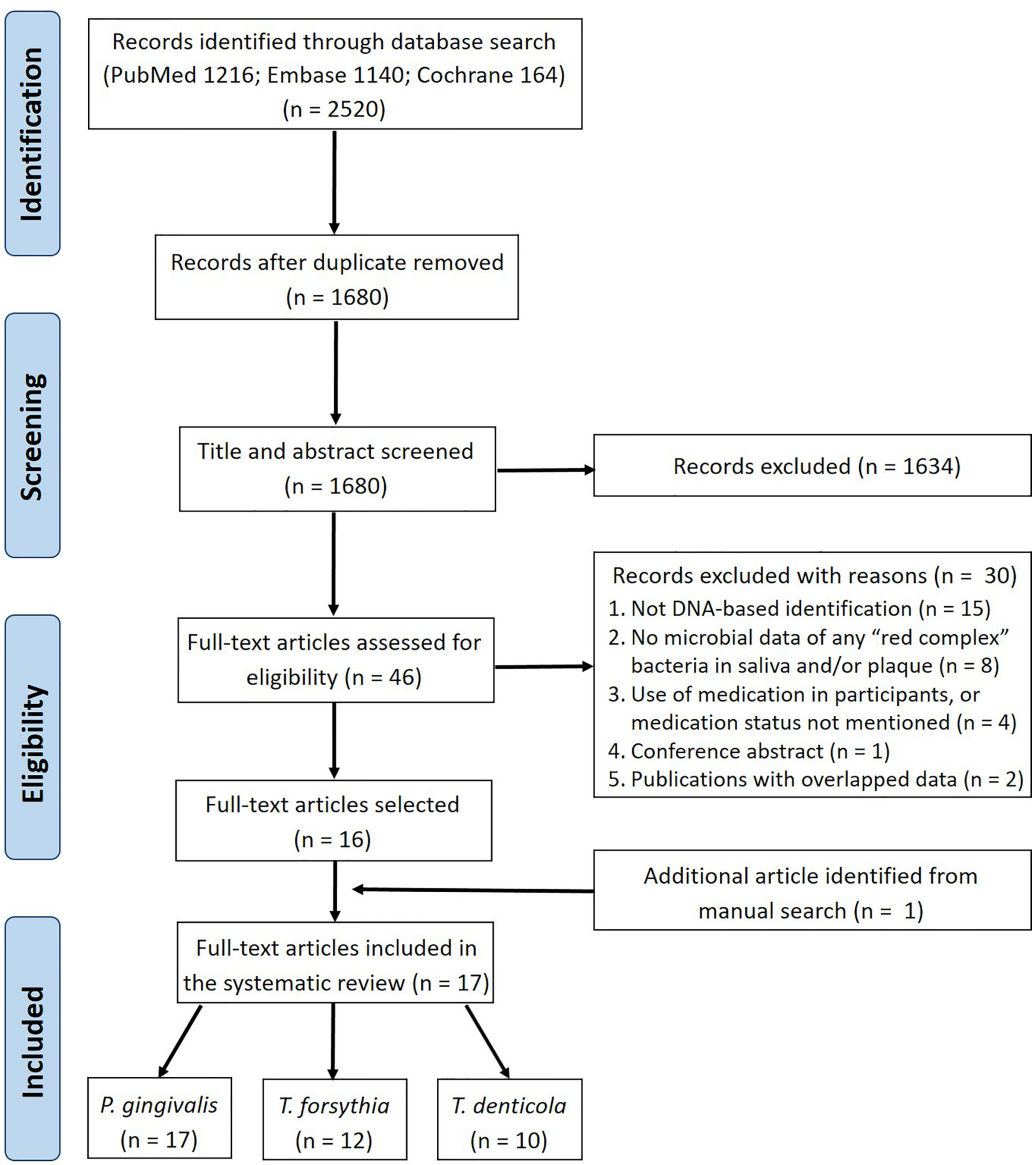
Flow diagram of the literature search and study selection.

The literature search of three electronic databases identified 2520 records in total (**Figure 1**). After removing duplicates, 1680 articles were retained for title and abstract screening, from which 1634 articles were excluded and the remaining 46 were assessed further in full-text reading. Of these 46 studies, 30 were excluded based on the eligibility criteria (**Supplementary Table S2**). One study (Umeda et al., 1998) was identified additionally from the manual search of the reference lists of the selected studies. Therefore, 17 studies were finally included in this systematic review.

### 3.2 Quality Assessment

**Figure 2:**
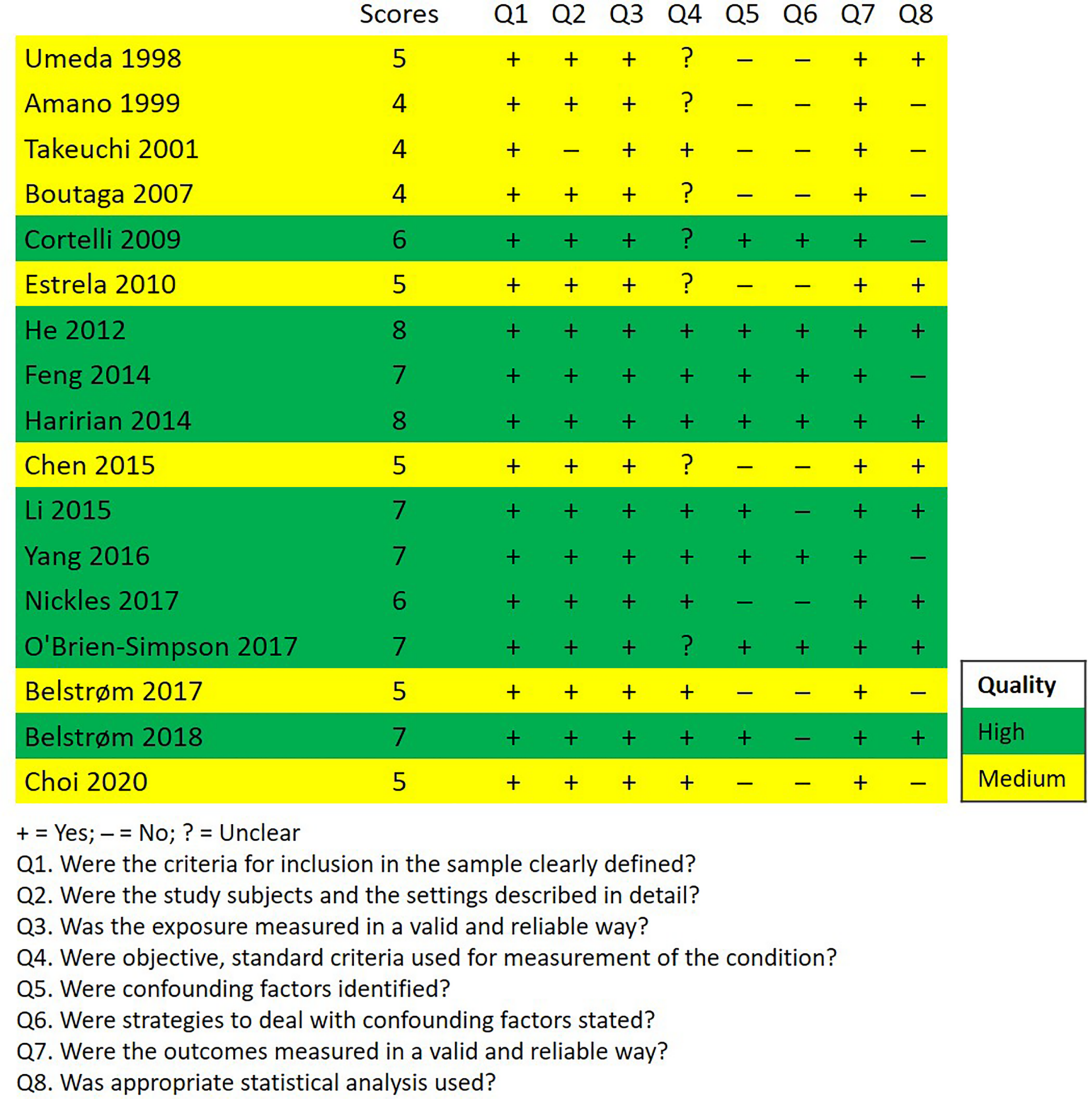
Quality assessment of the included studies according to the JBI Critical Appraisal Checklist for Analytical Cross-Sectional Studies.

**Figure 2** shows the methodological quality assessment of the included studies, which presented the answer to each appraisal criteria as well as an overall rating of each study. Nine out of 17 studies had high quality, 8 studies had medium quality and no study had low quality.

### 3.3 Characteristics of the Included Studies

The main characteristics of the included studies are presented in **Table 1**. Most studies had a cross-sectional design (n = 14); the rest had a prospective (n = 2) or interventional (n = 1) design.

**Table 1:** Main characteristics of all included studies.

Author (Country, Year)	Design	Patients	Diagnostic criteria	Saliva	Subgingival plaque			Method	Red complex reported
					Type	Sample site	Sample method		
Umeda et al. (USA, 1998)	Cross-sectional	CP (130); AgP (3)	NA	Unstimulated	Pooled	Deepest pockets	Paper point	PCR	*Pg; Tf; Td*
					(4 sites)				
Amano et al. (Japan, 1999)	Cross-sectional	P (93)	NA	Unstimulated	Pooled	Deepest pockets	Curette	PCR	*Pg*
					(2 sites)				
Takeuchi et al. (Japan, 2001)	Cross-sectional	CP (65); AgP (38)	International classification 1999*^a^*	Unstimulated	Individual	Deepest pockets	Paper point	PCR	*Pg; Td*
					(4 sites)				
Boutaga et al. (Netherlands, 2007)	Cross-sectional	P (21)	NA	Oral rinse	Pooled	Deepest pockets	Paper point	qPCR	*Pg; Tf*
					(4 sites)				
Cavalca Cortelli et al. (Brazil, 2009)	RCT	CP (20)	NA	Unstimulated	Pooled	Two sites of PPD ≥ 5 mm with BOP and CAL per quadrant	Paper point	PCR	*Pg; Tf*
					(8 sites)				
Estrela et al. (Brazil, 2010)	Cross-sectional	CP (30)	NA	Unstimulated	Pooled	NA	Paper point	Multiplex PCR	*Pg*
					(2 sites)				
He et al. (China, 2012)	Cross-sectional	CP (60)	≥ 4 teeth with BOP, CAL and radiographic alveolar bone loss, and PPD ≥ 4 mm in ≥ 4 sites not on the same tooth	Unstimulated	Pooled	Deepest pockets	Paper point	qPCR	*Pg*
					(4 sites)				
Feng et al. (China, 2014)	Cross-sectional	AgP (81)	International classification 1999*^a^*	Unstimulated	Pooled	Site of PPD ≥ 4 mm and CAL ≥ 2 mm of the first molars	Curette	PCR	*Pg; Tf; Td*
					(4 sites)				
Haririan et al. (Austria, 2014)	Cross-sectional	CP (43); AgP (33)	International classification 1999*^a^*	Oral rinse	Pooled	Deepest pockets	Paper point	microarray technique	*Pg; Tf; Td*
					(4 sites)				
Chen et al. (China, 2015)	Cross-sectional	P (30)	NA	Unstimulated	Pooled	Deepest pockets	Curette	16S rRNA gene sequencing	*Pg; Tf*
					(4 sites)			(454 GS FLX)	
Li et al. (China, 2015)	Cross-sectional	CP (10); AgP (10)	International classification 1999*^a^*	Unstimulated	Pooled	Site of PPD ≥ 4mm and CAL ≥ 2mm of the first molars	Curette	16S rRNA gene sequencing	*Pg; Tf; Td*
					(4 sites)			(454 GS FLX)	
Yang et al. (China, 2016)	Prospective	CP (45)	International classification 1999*^a^*	Unstimulated	Pooled	All teeth	Paper point	qPCR	*Pg; Tf; Td*
Nickles et al. (Germany, 2017)	Cross-sectional	CP (27); AgP (23)	Vertical CAL ≥ 5 mm at > 30% sites and age > 35 y (CP); clinically healthy, radiographic bone loss ≥ 50% on ≥ 2 different teeth and age < 35 y (AgP)	Oral rinse	Pooled	Deepest pockets	Paper point	qPCR	*Pg; Tf; Td*
					(4 sites)				
O’Brien-Simpson et al. (Australia, 2017)	Cross-sectional	CP (50)	NA	Stimulated	Individual	Deepest pockets	Curette	qPCR	*Pg*
					(6 sites)				
Belstrøm et al. (Denmark, 2017)	Cross-sectional	CP (18)	Task force report by the AAP*^b^*	Stimulated	Pooled; individual	Deepest pockets	Paper point	16S rRNA gene sequencing	*Pg; Tf; Td*
					(3 sites)*^c^*			(Illumin a MiSeq)	
Belstrøm et al. (Denmark, 2018)	Prospective	CP (24)	Task force report by the AAP*^b^*	Stimulated	Pooled	Deepest pockets	Curette	16S rRNA gene sequencing	*Pg; Tf; Td*
					(4 sites)			(Illumina MiSeq)	
Choi et al. (Korea, 2020)	Cross-sectional	MP (38); SP (38)*^d^*	Modification of CDC-AAP case definitions*^d^*	Oral rinse	Pooled	Deepest pockets	Paper point	qPCR	*Pg; Tf; Td*
					(3 sites)				

NA, not available.

RCT, randomized clinical trial; CP, chronic periodontitis; AgP, aggressive periodontitis; P, periodontitis unclassified; SP, severe periodontitis; MP, moderate periodontitis; PPD, probing pocket depth; CAL, clinical attachment loss; BOP, bleeding on probing; PCR, polymerase chain reaction; qPCR, quantitative real-time PCR; Pg, Porphyromonas gingivalis; Tf, Tannerella forsythia; Td, Treponema denticola.

a 1999 International Classification of Periodontal Diseases and Conditions.

b American Academy of Periodontology (AAP) Task Force Report on the Update to the 1999 Classification of Periodontal Diseases and Conditions.

c Only data from pooled subgingival plaque samples were used for analysis in this systematic review, in order to be comparable with data from other studies.

d Case definition introduced by the US Centers for Disease Control and Prevention and the American Academy of Periodontology (CDC-AAP). MP, moderate periodontitis; SP, severe periodontitis.

Out of the 17 studies, 14 studies reported the classification of periodontitis: 7 studies included patients with chronic periodontitis (CP) only, 1 study with aggressive periodontitis (AgP) only, and 5 studies with both CP and AgP. Different from the other 13 studies, Choi et al. (2020) classified the disease as moderate and severe periodontitis. Among these 14 studies, only 10 studies specified their diagnostic criteria: 5 studies (Takeuchi et al., 2001; Feng et al., 2014; Haririan et al., 2014; Li et al., 2015; Yang et al., 2016) followed the criteria of the 1999 International Classification of Periodontal Diseases and Conditions (Armitage, 1999), 2 studies (Belstrøm et al., 2017; Belstrøm et al., 2018) used the task force report by the American Academy of Periodontology (Geurs, 2015), 1 study (Choi et al., 2020) used a criteria modified from the case definition by the US Centers for Disease Control and Prevention and the American Academy of Periodontology (Page and Eke, 2007), and 2 studies (He et al., 2012; Nickles et al., 2017) used self-defined criteria. Three studies (Amano et al., 1999; Boutaga et al., 2007; Chen et al., 2015) did not specify the type of periodontitis and the diagnostic criteria.

For sample collection, saliva was collected as unstimulated (10 studies), stimulated (3 studies) or oral rinse sample (4 studies); subgingival plaque was collected by paper point in 11 studies and by curette in 6 studies. Most studies (n = 14) analyzed subgingival plaque samples pooled from multiple periodontal pockets (2 to full mouth). Only 2 studies analyzed plaque samples from individual pocket (Takeuchi et al., 2001; O’Brien-Simpson et al., 2017) and 1 study analyzed both pooled and individual plaque samples (Belstrøm et al., 2017).

With regard to the methods used for microbial identification, 4 studies used open-ended 16S rRNA gene sequencing techniques (Chen et al., 2015; Li et al., 2015; Belstrøm et al., 2017; Belstrøm et al., 2018), and the remaining 13 studies used targeted PCR-based approaches, including PCR, qPCR, and microarray techniques.

### 3.4 Clinical Data

The most frequently reported clinical data in the included studies, mean probing pocket depth (PPD), clinical attachment loss (CAL) and bleeding on probing (BOP) of full mouth and/or the sampled sites, are summarized in **Table 2**. Two studies did not report any clinical data (Amano et al., 1999; Estrela et al., 2010). Overall, there were no big variations among the reported mean PPD and/or CAL, except that 2 studies reported a mean PPD less than 3 mm (He et al., 2012; Choi et al., 2020). The mean PPD of the sampled sites generally ranged from 4 to 8 mm.

**Table 2:** Clinical parameters (PPD, CAL and BOP) of periodontitis patients in the included studies.

Study	Classification of Periodontitis	Full mouth			Sampled sites		
		Mean PPD	Mean CAL	Mean BOP	Mean PPD	Mean CAL	Mean BOP
		(mm)	(mm)	(% sites)	(mm)	(mm)	(% sites)
Umeda et al., 1998	CP	–	–	–	5.10 ± 1.50	–	–
	AgP	–	–	–	5.60 ± 0.70	–	–
Amano et al., 1999	P	–	–	–	–	–	–
Takeuchi et al., 2001	CP	–	–	–	5.82 ± 2.21	6.66 ± 2.51	57.30
	AgP	–	–	–	5.84 ± 2.40	6.20 ± 2.70	77.60
Boutaga et al., 2007	P	–	–	–	6.48 ± 1.04	4.45 ± 3.68	–
Cavalca Cortelli et al., 2009	CP	4.96 ± 0.48	–	–	–	–	–
Estrela et al., 2010	CP	–	–	–	–	–	–
He et al., 2012	CP	2.70 ± 0.70	2.40 ± 1.80	41.00 ± 18.70	3.90 ± 1.30	3.90 ± 2.70	–
Feng et al., 2014	AgP	5.02 ± 1.08	4.67 ± 1.53	–	6.85 ± 1.47	6.03 ± 1.86	–
Haririan et al., 2014	CP	4.01 ± 0.93	4.54 ± 1.21	40.82 ± 23.64	7.19 ± 1.12	–	–
	AgP	3.87 ± 0.91	4.39 ± 0.95	46.22 ± 24.82	7.52 ± 1.13	–	–
Chen et al., 2015	P	4.80 ± 0.96	4.30 ± 1.43	–	–	–	–
Li et al., 2015	CP	4.50 ± 1.24	4.40 ± 1.05	100	5.47 ± 1.24	5.85 ± 2.47	–
	AgP	4.84 ± 0.91	4.28 ± 1.33	100	6.95 ± 0.74	5.92 ± 0.91	–
Yang et al., 2016	CP	3.21 ± 0.86	2.09 ± 1.32	–	–	–	–
Nickles et al., 2017	CP	–	–	–	8.61 ± 1.32	8.99 ± 1.28	–
	AgP	–	–	–	7.96 ± 1.97	8.15 ± 2.40	–
O’Brien-Simpson et al., 2017	CP	3.60 ± 1.00	7.00 ± 2.10	60.80 ± 25.30	–	–	–
Belstrøm et al., 2017	CP	–	–	–	7.00	8.00	–
Belstrøm et al., 2018	CP	3.40	4.10	56.00	6.40	7.00	–
Choi et al., 2020	MP	2.49	2.65	47.13	–	–	–
	SP	2.89	3.82	53.91	–	–	–

Data are presented as mean or mean ± SD of full mouth and/or sampled sites where the subgingival plaque samples were collected.

PPD, probing pocket depth; CAL, clinical attachment loss; BOP, bleeding on probing; CP, chronic periodontitis; AgP, aggressive periodontitis; P, periodontitis unclassified; MP, moderate periodontitis; SP, severe periodontitis.

### 3.5 Microbial Data

#### 3.5.1 Detection Frequency of the Red-Complex Bacteria

**Figure 3:**
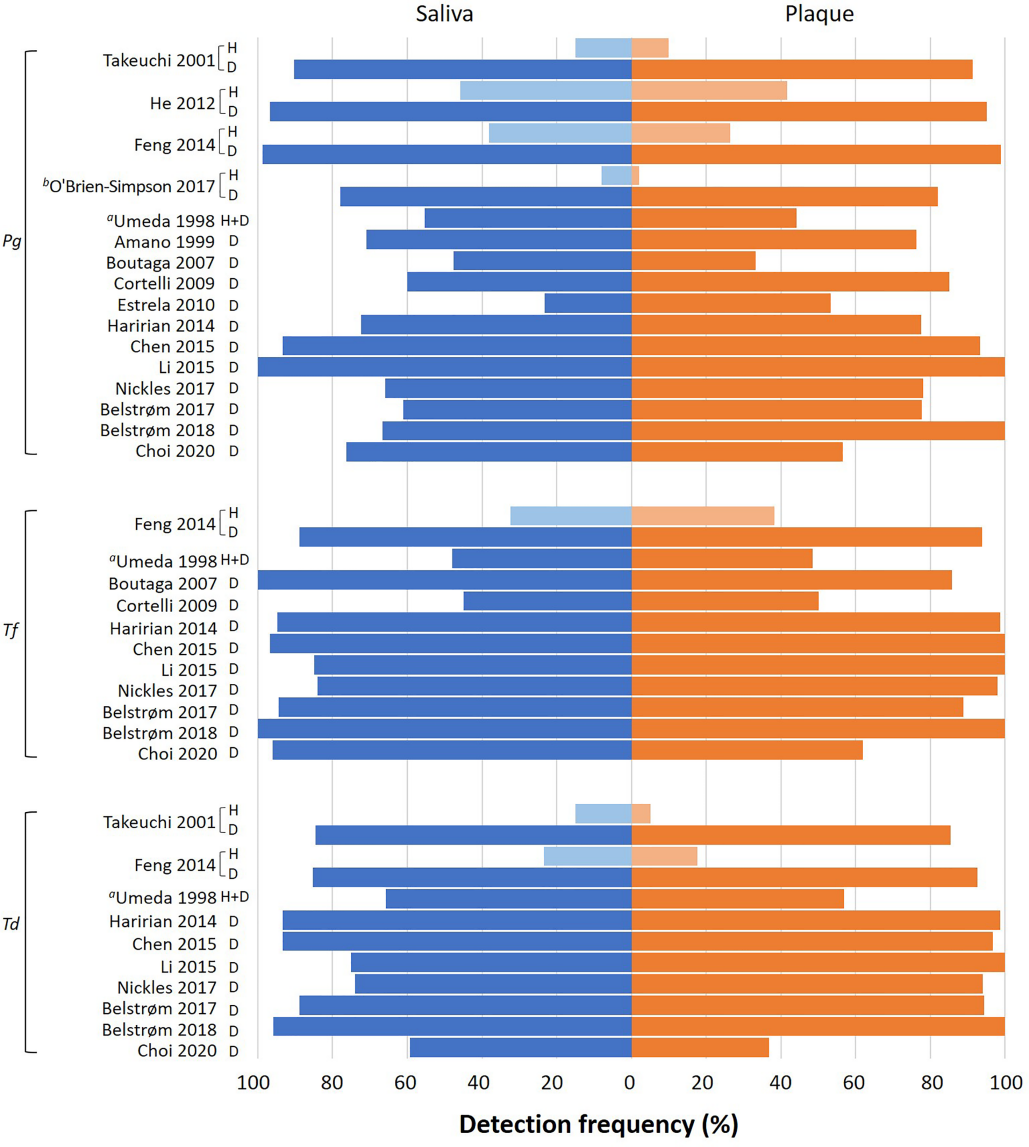
Overview of the detection frequency of *P. gingivalis* (*Pg*), *T. forsythia* (*Tf*) and *T. denticola* (*Td*) in saliva and subgingival plaque samples reported in each study. H: healthy subjects; D: periodontitis patients. Pooled subgingival plaque samples were used for analysis unless specified otherwise. *^a^* This study mentioned 3 groups of subjects (health, gingivitis and periodontitis), but the data of different subject groups were reported together. *^b^* This study analyzed the subgingival plaque samples from 6 periodontal pockets individually.

The detection frequency of at least one of the red-complex bacteria could be extracted from 16 out of the 17 included studies. One study (Yang et al., 2016) did not report detection frequency, but bacterial counts only. **Figure 3** presents an overview of bacterial detection frequencies in saliva and subgingival plaque samples. Most studies reported the detection frequencies of the red-complex bacteria were more than 60% in both saliva and subgingival samples of periodontitis patients. The only study which reported detection frequencies of less than 60% for all 3 species combined the data of healthy subjects and periodontitis patients (Umeda et al., 1998). Four studies also included healthy subjects, which showed varied detection frequencies of the red-complex bacteria, ranging from 2% to 45%. However, in each study, the detection frequencies of the red-complex bacteria in healthy group were much lower than those in the corresponding periodontitis group, irrespective of the sample types (Takeuchi et al., 2001; He et al., 2012; Feng et al., 2014; O’Brien-Simpson et al., 2017). Generally, most studies reported that the detection frequency of a specific red-complex species in periodontitis patients was lower in saliva than in subgingival plaque. But a few studies reported opposite trends. These studies are Boutaga et al. (2007) and Choi et al. (2020) for *P. gingivalis*, Boutaga et al. (2007), Choi et al. (2020) and Belstrøm et al. (2017) for *T. forsythia*, and Choi et al. (2020) for *T. denticola*.

**Figure 4:**
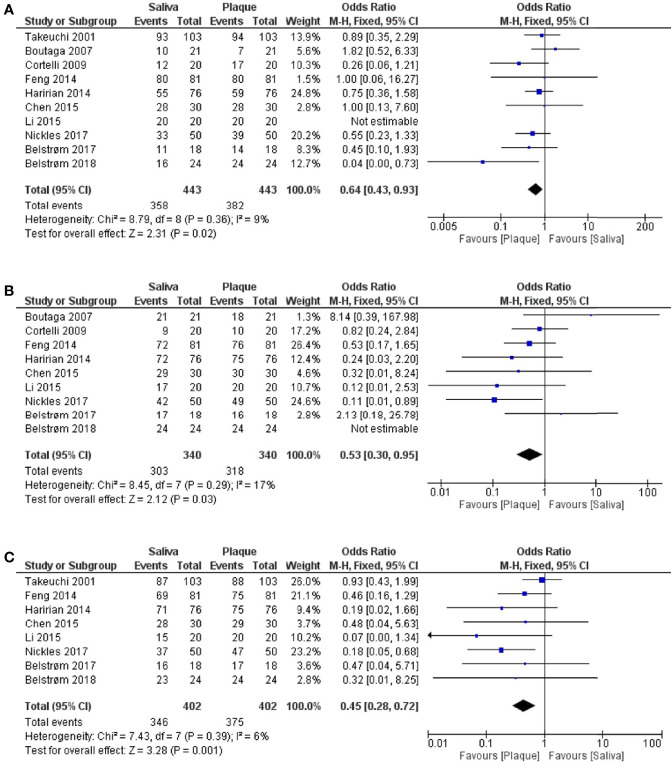
Forest plots of meta-analyses comparing the detection frequency of: **(A)** *P. gingivalis*; **(B)** *T. forsythia*; **(C)** *T. denticola* between saliva and subgingival plaque samples from patients with periodontitis.

Next, meta-analyses were performed by summarizing the results from different studies, in order to assess the differences between saliva and subgingival plaque samples statistically. In total, 10 out of 16 studies were included in the meta-analyses. Six studies were excluded due to the following reasons: 1. Umeda et al. (1998) did not report data per healthy, gingivitis and periodontitis group. Only the average data of 3 groups were given; 2. O’Brien-Simpson et al. (2017) analyzed subgingival plaque samples from 6 periodontal pockets individually while other studies used pooled subgingival plaque samples; 3. Since the pocket depth was believed to affect the profile of subgingival microbiota (Van Dyke et al., 2020), studies which did not report PPD (Amano et al., 1999; Estrela et al., 2010) and reported PPD < 3 mm (He et al., 2012; Choi et al., 2020) were excluded. As shown in **Figure 4**, meta-analyses revealed that the heterogeneity among studies was low for all 3 red-complex bacteria, with *I^2^* ranging from 6% to 17%. The detection frequencies of *P. gingivalis* [**Figure 4A**; OR = 0.64, 95% CI: (0.43, 0.93), *p* = 0.02], *T. forsythia* [**Figure 4B**; OR = 0.53, 95% CI: (0.30, 0.95), *p* = 0.03], and *T. denticola* [**Figure 4C**; OR = 0.45, 95% CI: (0.28, 0.72), *p* = 0.001] were all significantly higher in subgingival plaque samples than that in saliva samples.

#### 3.5.2 Bacterial Counts

Six out of the 17 studies reported bacterial counts of at least one of the red-complex species. Meta-analyses could not be performed on the data of bacteria counts due to the varied data format reported among studies (e.g., bacterial cell numbers and bacterial DNA copy numbers). Therefore, only a descriptive summary of the data is presented. As shown in **Table 3**, 4 studies (Boutaga et al., 2007; Yang et al., 2016; Nickles et al., 2017; O’Brien-Simpson et al., 2017) reported higher red-complex counts in subgingival plaque than in saliva, while the other 2 studies reported the opposite results (He et al., 2012; Choi et al., 2020). Only 1 study (Nickles et al., 2017) performed statistical analysis to confirm the reported higher counts in subgingival plaque.

**Table 3:** Comparisons of bacterial counts of the red complex species between saliva and subgingival plaque samples from periodontitis patients.

Study	*P. gingivalis*		*T. forsythia*		*T. denticola*	
	saliva *vs* plaque		saliva *vs* plaque		saliva *vs* plaque	
He et al., 2012	↑		–	–	–	–
O’Brien-Simpson et al., 2017		↑	–	–	–	–
Boutaga et al., 2007		↑		↑	–	–
Yang et al., 2016		↑		↑		↑
Nickles et al., 2017		↑^*^		↑^*^		↑^*^
Choi et al., 2020	↑		↑		↑	

↑Higher in the corresponding sample.

*Data presented in the study were evaluated statistically.

#### 3.5.3 Relative Abundance of the Red-Complex Bacteria

The relative abundance data were extracted from 4 sequencing studies in the following ways: from the published data (Belstrøm et al., 2017), from the data provided by the authors upon request (Li et al., 2015; Belstrøm et al., 2018), and from the raw sequence data that have been uploaded to the Sequence Read Archive (Chen et al., 2015).

**Figure 5:**
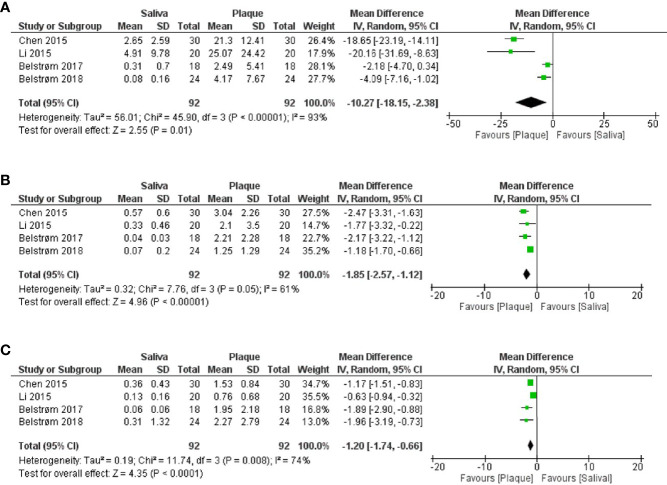
Forest plots of meta-analyses comparing the relative abundances (%) of: **(A)** *P. gingivalis*; **(B)** *T. forsythia*; **(C)** *T. denticola* between saliva and subgingival plaque samples from patients with periodontitis, as determined in studies using 16S rRNA gene sequencing techniques.

As shown in **Figure 5**, meta-analyses revealed that the relative abundances of *P. gingivalis* [**Figure 5A**; MD = -10.27, 95% CI: (-18.15, -2.38), *p* < 0.00001], *T. forsythia* [**Figure 5B**; MD = -1.85, 95% CI: (-2.57, -1.12), *p* < 0.00001] and *T. denticola* [**Figure 5C**; MD = -1.20, 95% CI: (-1.74, -0.66), *p* < 0.0001] in subgingival plaque were all significantly higher than in saliva. However, considerably high heterogeneities among 4 studies were observed for the data of all 3 bacteria. Taking *P. gingivalis* as an example, the reported mean relative abundance ranged from 2.5 to 25% in subgingival plaque and from 0.1% to 5% in saliva, with an *I^2^* index of 93%.

**Figure 6:**
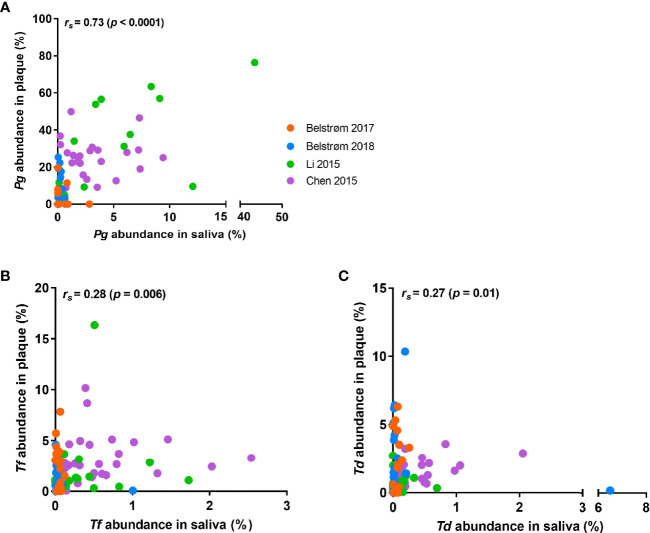
Scatter plots showing the distributions of the relative abundance of: **(A)** *P. gingivalis*; **(B)** *T. forsythia*; **(C)** *T. denticola* in saliva and subgingival plaque samples per patient, as determined in studies using 16S rRNA gene sequencing techniques. Each dot represents one patient, and patients from different studies are indicated by different colors of the dots. Spearman’s rank correlation coefficient (*r_s_*) and *p* value are shown in each plot.

Since the relative abundance data of each patient were available from all 4 sequencing studies, we conducted Spearman’s rank correlation analysis in SPSS version 25 (SPSS Inc., Chicago, IL, USA) using the paired data obtained from saliva and subgingival plaque per patient. **Figure 6** shows that positive correlations in relative abundance between saliva and subgingival plaque samples were observed for all 3 red-complex species, with a correlation coefficient of 0.73 for *P. gingivalis* (*p* < 0.0001), 0.28 for *T. forsythia* (*p* = 0.006) and 0.27 for *T. denticola* (*p* = 0.01).

#### 3.5.4 Predominant Bacterial Genera in Saliva and Plaque Samples

**Figure 7:**
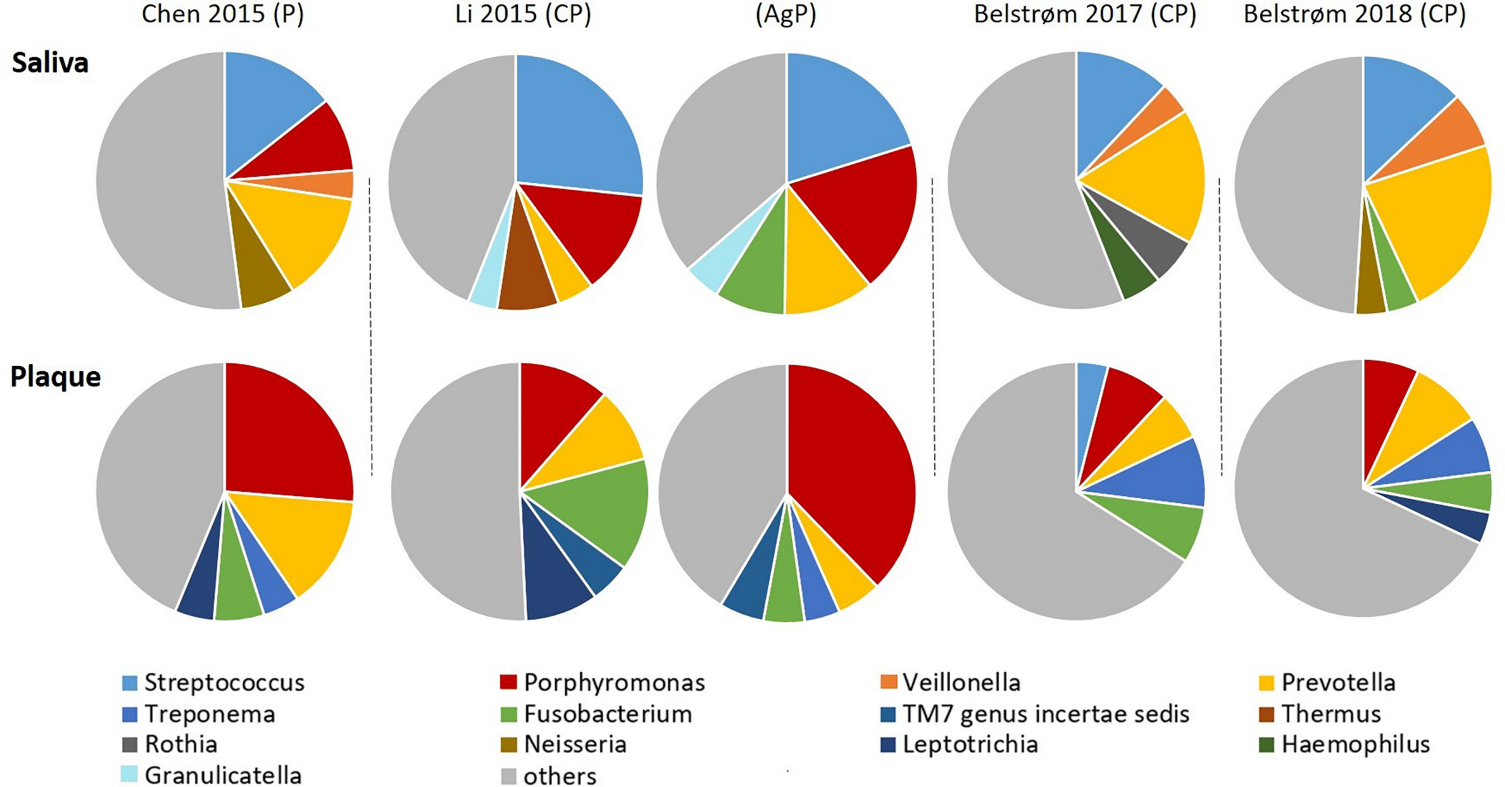
Approximate relative abundance of the top 5 most abundant genera identified in saliva and subgingival plaque from patients with periodontitis, as determined in studies using 16S rRNA gene sequencing techniques. CP, chronic periodontitis; AgP, aggressive periodontitis; P, periodontitis unclassified.

The open-ended sequencing techniques allow the detection of more bacterial species than the targeted approach. Hence, the microbial compositions were summarized from 4 sequencing studies. The top 5 most abundant bacterial genera of each study are shown in **Figure 7**. Within each study, the top 5 most abundant bacterial genera in saliva were generally different from those in subgingival plaque, suggesting a major compositional difference between these two sample types. Among the most abundant genera, 4 bacterial genera were shared by all 4 studies: *Streptococcus* and *Prevotella* in saliva samples and *Porphyromonas* and *Fusobacterium* in subgingival plaque samples.

## 4 Discussion

It has been widely accepted that periodontitis is an inflammatory disease caused by a dysbiotic microbial community (Hajishengallis, 2015). Representative microbial sampling is crucial for disease prevention, diagnosis and treatment. Subgingival plaque in periodontal pockets represents the onset and development of periodontitis the best. However, due to its complicated sampling process, the use of saliva as an alternative has been an interest in many clinical studies. This systematic review identified 17 studies that reported the levels of red-complex bacteria in both saliva and subgingival plaque in periodontitis patients. Three types of outcome parameters, detection frequency, bacterial count and relative abundance, were examined. The meta-analyses on both detection frequency and relative abundance revealed that the levels of the red-complex bacteria in saliva were significantly lower than those in subgingival plaque.

Previously various researchers have claimed, based on their own data, that saliva could be a potential alternative to subgingival plaque for microbiologic analysis in periodontitis patients (Boutaga et al., 2007; Haririan et al., 2014; Belstrøm et al., 2017). Our meta-analysis summarized the results based on the samples obtained from 443 periodontitis patients in 10 studies (**Figure 4**). We found that saliva samples cannot represent the levels (i.e., detection frequency and relative abundance) of red-complex bacteria in subgingival plaque accurately in these patients. Since all the data analyzed in this review were obtained from samples taken at one time point, our finding indicates that one-time saliva sampling cannot be used to screen patients for the red-complex bacteria. We also examined other factors which might influence the comparison between saliva and subgingival plaque samples. Interestingly, the subgroup analysis (**Supplementary Material**; **Figure S1**) based on the collection methods of subgingival plaque, paper point or curette, showed that the results of studies using curette were in line with the finding mentioned above. However, in the studies using paper point, the detection frequencies of *P. gingivalis* and *T. forsythia* in saliva and subgingival plaque samples were similar. Possibly, a paper point collects unattached microbes in the periodontal pocket, which are likely spilt over to saliva; whereas a curette collects firmly attached biofilms (Jervøe-Storm et al., 2007). Clinical findings on the collection methods of subgingival plaque are inconsistent: one study (Renvert et al., 1992) stated that paper point sampling presented different microbial information as compared to curette sampling; whereas another study claimed a good agreement for the results of two sampling methods. Moreover, the open-ended sequencing method revealed DNA contamination in paper points, making this collection method unsuitable for sequencing analysis (van der Horst et al., 2013). Taken together, the methods used for collecting subgingival plaque may potentially influence microbial composition comparisons between saliva and subgingival plaque.

It is worth noting that our data analyses demonstrated positive associations between saliva and subgingival plaque in terms of red-complex levels despite limited data. Among the 17 included studies, 4 studies not only examined samples from periodontitis groups, but also from periodontal healthy groups. Although the reported detection frequencies in the healthy subjects varied considerably among studies (2% to 45%), all 4 studies showed that within one study, the detection frequency of each red-complex bacteria was much higher in periodontitis group than that in healthy group, irrespective of the sample type. Moreover, the relative abundances of the red-complex bacteria in saliva were also significantly correlated to that in subgingival plaque (**Figure 6**). Hence, it is possible that once the red-complex bacteria are enriched in subgingival plaque at diseased state, they could be spilt over or washed out into saliva, which consequently increase their levels in saliva. To this end, sampling saliva at multiple time points might help to trace the compositional shift of subgingival microbiota towards a disease provoking state. The study of Belstrøm et al. (2018) showed the correlation of the levels of salivary red-complex bacteria to that of subgingival plaque before and after treatment, indicating the possibility of such application of saliva sample. However, a thorough review on longitudinal clinical studies is needed to confirm this.

The magnitude and presence of statistical heterogeneity in a meta-analysis are usually explained by heterogeneity of methodological and/or clinical sources (Deeks et al., 2021). In our results, high heterogeneities of the relative abundance data among the 4 sequencing studies were observed, irrespective of the targeted bacterial species (*I^2^*: 61%–93%). Since the sequencing method-related heterogeneity in the meta-analysis of microbiome data has been reported before (Lozupone et al., 2013; Duvallet, 2018), the source of high heterogeneity here is likely related to the sequencing techniques. All 4 studies varied in many steps in sample processing as well as analysis, such as the DNA extraction methods, targeted 16S rRNA gene region for sequencing, sequencing platform used and sequencing depth. Lozupone et al. (2013) conducted meta-analysis on human microbiome data extracted from 12 different studies, and revealed that the technical variations in different studies could obscure biologically meaningful compositional differences. However, when the studied parameter had a large effect size (e.g., the body sites), the bias caused by variation in sequencing methodology could be outweighed by the real difference. In our case, despite the high heterogeneity, the relative abundance of red-complex bacteria in saliva was consistently lower than that in subgingival plaque.

During data analysis, we identified a clinical parameter, periodontal pocket depth, which potentially contributed to the high heterogeneity in a meta-analysis. In addition to the results on the basis of 10 studies reported in **Figure 4**, we also performed a meta-analysis on the detection frequencies reported in 14 studies, where an additional 4 studies with unknown or low periodontal pocket depth were included (**Supplementary Material**; **Figure S2**). From these meta-analyses, we observed substantially high heterogeneities (*I^2^*: 49%–73%) as compared to those in the 10-study based meta-analyses (*I^2^*: 6%–17%). The 14-study based meta-analyses showed no significant difference in the detection frequency of all 3 red-complex bacteria between saliva and subgingival plaque. We suspected that the high heterogeneity of the 14-study based meta-analyses was, at least partly, caused by variations in pocket depths. Van Dyke et al. (2020) stated that the depth of periodontal pocket was one of the crucial elements which determined the dysbiosis of subgingival microbiota, as differentiate microbial profiles were observed in pockets with different depths. For example, the abundance of *Bacteriodetes* significantly increased as the pocket deepened (Kirst et al., 2015). Our observation showed that the periodontal pocket depth might be an important confounding factor for microbial analysis. Interestingly, in the summary of bacterial count data, the 2 studies that reported a low pocket depth were also the only 2 studies which showed higher salivary red-complex bacterial counts in saliva as compared to subgingival plaque (He et al., 2012; Choi et al., 2020). Likely, the differential levels of red-complex bacteria in saliva and subgingival plaque are associated with the depth of periodontal pockets. Unfortunately, most studies included this review obtained samples from deep pockets, and we could not further illustrate the potential influence of this confounding factor.

In conclusion, this systematic review shows that the levels of red-complex bacteria in saliva were significantly lower than those in subgingival plaque in patients with periodontitis, in terms of the detection frequency and relative abundance. This finding is based on the meta-analyses on the data obtained from 443 patients at one sampling time point. In addition, our analyses reveal positive associations in the levels of red-complex bacteria between saliva and subgingival plaque despite limited data. Therefore, we recommend a thorough review of longitudinal clinical studies to further assess the role of saliva in detecting periodontitis-related microorganisms.

## Data Availability Statement

The original contributions presented in the study are included in the article/**Supplementary Material**. Further inquiries can be directed to the corresponding authors.

## Author Contributions

YJ and DD conceived and designed the study. YJ and BS performed the study and collected the data. YJ, BB, and DD analyzed and interpreted the data. YJ drafted the manuscript. BB, LC, XZ, RE, WC, and DD revised the manuscript critically. All authors contributed to the article and approved the submitted version.

## Funding

This study was supported by the ACTA Research Institute and the National Natural Science Foundation of China grant 81870759 (to LC).

## Conflict of Interest

The authors declare that the research was conducted in the absence of any commercial or financial relationships that could be construed as a potential conflict of interest.

## Publisher’s Note

All claims expressed in this article are solely those of the authors and do not necessarily represent those of their affiliated organizations, or those of the publisher, the editors and the reviewers. Any product that may be evaluated in this article, or claim that may be made by its manufacturer, is not guaranteed or endorsed by the publisher.
