# Supplementary material for: Comparison of Red-Complex Bacteria Between Saliva and Subgingival Plaque of Periodontitis Patients: A Systematic Review and Meta-Analysis
Source: Front Cell Infect Microbiol. 2021 Oct 8;11:727732. doi: 10.3389/fcimb.2021.727732 (PMC8531218; doi:10.3389/fcimb.2021.727732)
Supplement: Supplementary file 1 [file DataSheet_1.docx]

Supplementary Materials

# Supplementary Tables and Figures

## Supplementary Tables

**Table S1.** The search strategy used in this systematic review.

| **Database** | **Search Strategies** |
| --- | --- |
| PubMed | ((((periodontal disease) OR (periodontitis)) OR ("periodontal infection")) AND ((((((((((((16S rRNA) OR (sequencing)) OR (microbiome)) OR (microbiota)) OR ("microbial community")) OR (Bacteroidetes)) OR (Spirochaetes)) OR ("red complex")) OR (Porphyromonas gingivalis)) OR (Tannerella forsythia)) OR (Treponema denticola)) OR (periopathogen))) AND (((saliva) OR (salivary)) OR (mouthwash)) |
| EMBASE | #1 periodontal disease'/exp OR 'periodontal disease'  #2 periodontitis'/exp OR 'periodontitis'  #3 periodontal infection'  #4 #1 OR #2 OR #3  #5 '16s rrna'/exp OR '16s rrna'  #6 'sequencing'/exp OR sequencing  #7 'microbiome'/exp OR 'microbiome'  #8 'microbiota'/exp OR microbiota  #9 'microbial community'/exp OR 'microbial community'  #10 bacteroidetes'/exp OR 'bacteroidetes'  #11 'spirochaetes'/exp OR spirochaetes  #12 'red complex'  #13 'porphyromonas gingivalis'/exp OR 'porphyromonas gingivalis'  #14 'tannerella forsythia'/exp OR 'tannerella forsythia'  #15 treponema denticola'/exp OR 'treponema denticola'  #16 periopathogen  #17 #5 OR #6 OR #7 OR #8 OR #9 OR #10 OR #11 OR #12 OR #13 OR #14 OR #15 OR #16  #18 'saliva'/exp OR saliva  #19 salivary  #20 'mouthwash'/exp OR 'mouthwash'  #21 #18 OR #19 OR #20  #22 #4 AND #17 AND #21 |
| Cochrane | #1 MeSH descriptor: [Periodontal Diseases] explode all trees  #2 periodontal disease  #3 MeSH descriptor: [Periodontitis] explode all trees  #4 periodontitis  #5 periodontal infection  #6 (#1 OR #2 OR #3 OR #4 OR #5)  #7 MeSH descriptor: [RNA, Ribosomal, 16S] explode all trees  #8 16S rRNA  #9 sequencing  #10 MeSH descriptor: [Microbiota] explode all trees  #11 microbiome  #12 microbiota  #13 MeSH descriptor: [Bacteroidetes] explode all trees  #14 Bacteroidetes  #15 MeSH descriptor: [Spirochaeta] explode all trees  #16 Spirochaetes  #17 red complex  #18 MeSH descriptor: [Porphyromonas gingivalis] explode all trees  #19 Porphyromonas gingivalis  #20 MeSH descriptor: [Tannerella forsythia] explode all trees  #21 Tannerella forsythia  #22 MeSH descriptor: [Treponema denticola] explode all trees  #23 Treponema denticola  #24 periopathogen  #25 (#7 OR #8 OR #9 OR #10 OR #11 OR #12 OR #13 OR #14 OR #15 OR #16 OR #17 OR #18 OR #19 OR #20 OR #21 OR #22 OR #23 OR #24)  #26 MeSH descriptor: [Saliva] explode all trees  #27 saliva  #28 salivary  #29 MeSH descriptor: [Mouthwashes] explode all trees  #30 mouthwash  #31 (#26 OR #27 OR #28 OR #29 OR #30)  #32 (#6 AND #25 AND #31) |

**Table S2.** Reasons for exclusion after full text reading.

| **Exclusion reason** | **Study** |
| --- | --- |
| Bacterial identification method was not DNA-based | Zambon et al., 1981; Van Winkelhoff et al., 1988; van Steenbergen et al., 1993; Petit et al., 1994; Nieminen et al., 1995; Von Troil-Lindén et al., 1995; Danser et al., 1996; von Troil-Lindén et al., 1996; Bollen et al., 1998; Timmerman et al., 1998; Quirynen et al., 1999; Quirynen et al., 2000; Timmerman et al., 2000; Lakio et al., 2009; Xu et al., 2018 |
| No microbial data of any red-complex bacteria reported in saliva and/or plaque samples, or data were only presented in figure format | Saygun et al., 2011; Huang et al., 2014; Kageyama et al., 2017; Noguera-Julian et al., 2017; Chen et al., 2018; Almeida et al., 2020; Li et al., 2020; Zhang et al., 2021 |
| Participants used medication, or medication status was not mentioned | Beikler et al., 2004; Beikler et al., 2006; Quirynen and Van Assche, 2011; de Waal et al., 2014 |
| Conference abstract | O'Brien-Simpson et al., 2015 |
| Publications with overlapped data | He et al., 2013; Feng et al., 2015 |

## Supplementary Figures

**
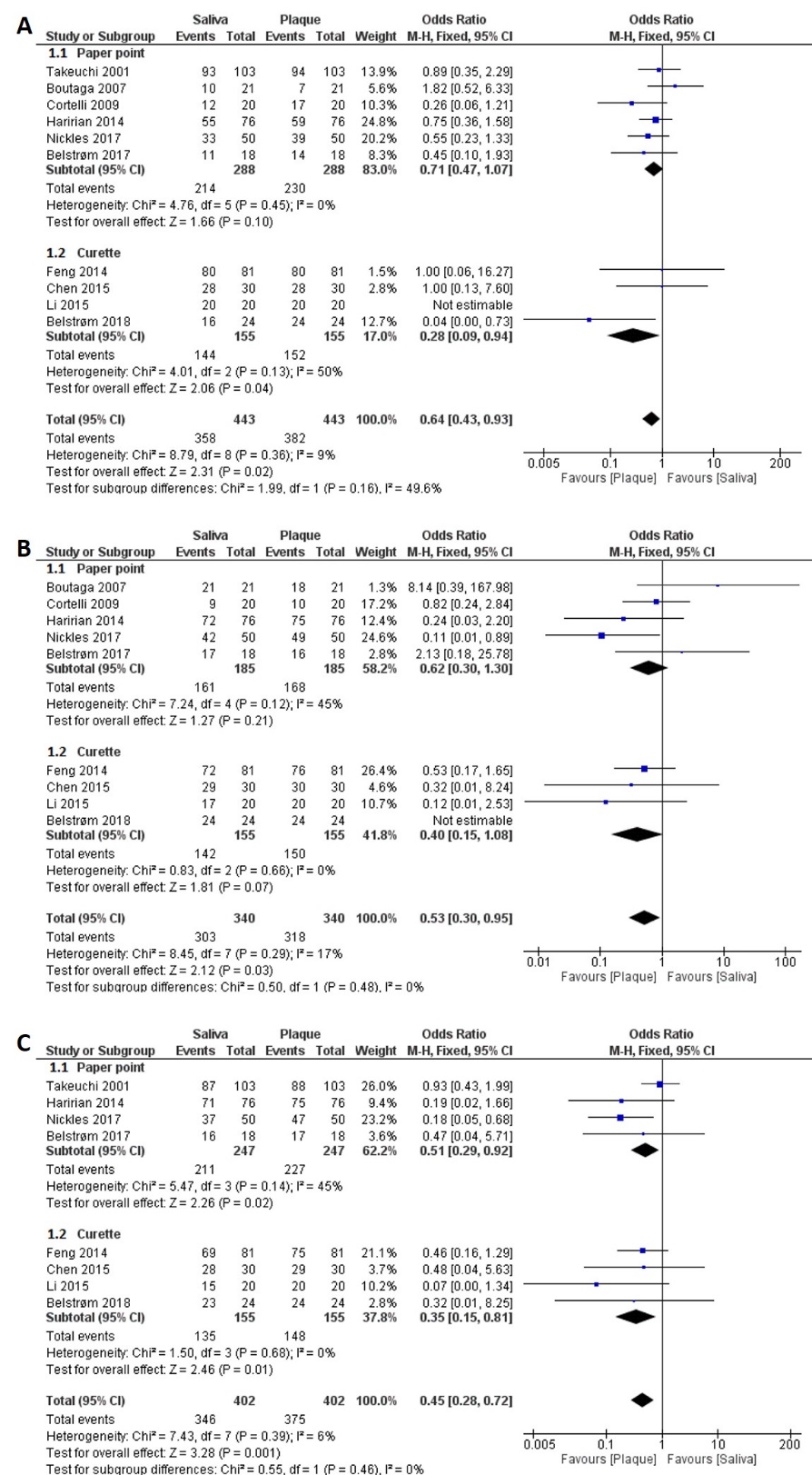
**

**Figure S1.** Forest plots of subgroup meta-analyses based on the collection methods of subgingival plaque (paper point or curette), comparing the detection frequencies of: (A) *P. gingivalis*; (B) *T. forsythia*; (C) *T. denticola* between saliva and subgingival plaque samples taken from patients with periodontitis.

**
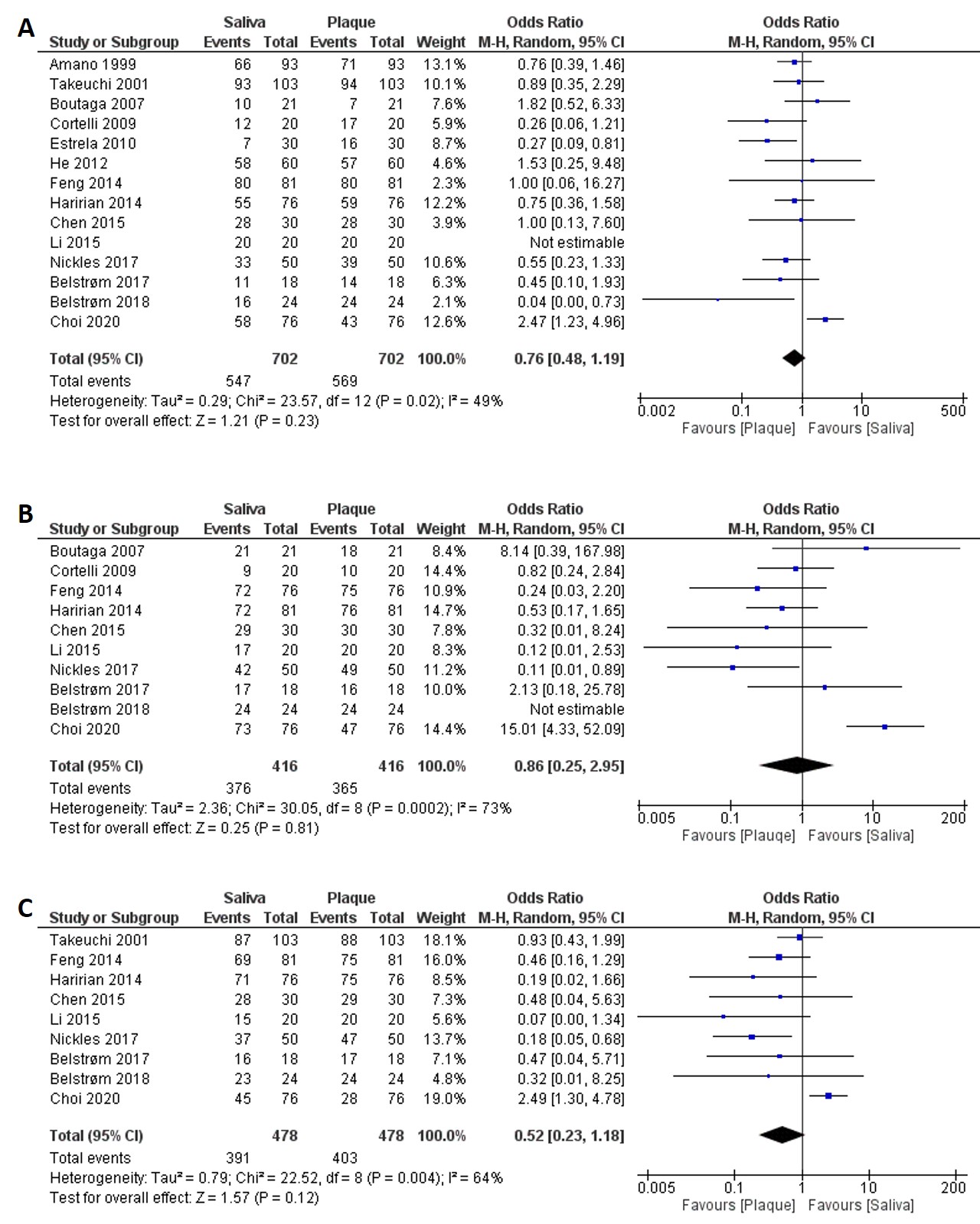
**

**Figure S2.** Forest plots of meta-analyses comparing the detection frequency of: (A) *P. gingivalis*; (B) *T. forsythia*; (C) *T. denticola* between saliva and subgingival plaque samples taken from patients with periodontitis. Data of all 14 studies were used for analysis, regardless of the reported or the absence of clinical data.

**References**

Almeida, V.S.M., Azevedo, J., Leal, H.F., Queiroz, A.T.L., da Silva Filho, H.P., and Reis, J.N. (2020). Bacterial diversity and prevalence of antibiotic resistance genes in the oral microbiome. *PLoS One* 15(9)**,** e0239664. doi: 10.1371/journal.pone.0239664.

Beikler, T., Abdeen, G., Schnitzer, S., Sälzer, S., Ehmke, B., Heinecke, A., et al. (2004). Microbiological shifts in intra- and extraoral habitats following mechanical periodontal therapy. *J. Clin. Periodontol.* 31(9)**,** 777-783. doi: 10.1111/j.1600-051X.2004.00557.x.

Beikler, T., Schnitzer, S., Abdeen, G., Ehmke, B., Eisenacher, M., and Flemmig, T.F. (2006). Sampling Strategy for Intraoral Detection of Periodontal Pathogens Before and Following Periodontal Therapy. *J. Periodontol.* 77(8)**,** 1323-1332. doi: 10.1902/jop.2006.050204.

Bollen, C.M.L., Mongardini, C., Papaioannou, W., Van Steenberghe, D., and Quirynen, M. (1998). The effect of a one-stage full-mouth disinfection on different intra-oral niches Clinical and microbiological observations. *J. Clin. Periodontol.* 25(1)**,** 56-66. doi: 10.1111/j.1600-051X.1998.tb02364.x.

Chen, C., Hemme, C., Beleno, J., Shi, Z.J., Ning, D., Qin, Y., et al. (2018). Oral microbiota of periodontal health and disease and their changes after nonsurgical periodontal therapy. *ISME. J.* 12(5)**,** 1210-1224. doi: 10.1038/s41396-017-0037-1.

Danser, M.M., Timmerman, M.F., van Winkelhoff, A.J., and van der Yelden, O. (1996). The Effect of Periodontal Treatment on Periodontal Bacteria on the Oral Mucous Membranes. *J. Periodontol.* 67(5)**,** 478-485. doi: 10.1902/jop.1996.67.5.478.

de Waal, Y.C.M., Winkel, E.G., Raangs, G.C., van der Vusse, M.L., Rossen, J.W.A., and van Winkelhoff, A.J. (2014). Changes in oral microflora after full-mouth tooth extraction: a prospective cohort study. *J. Clin. Periodontol.* 41(10)**,** 981-989. doi: 10.1111/jcpe.12297.

Feng, X., Zhu, L., Xu, L., Meng, H., Zhang, L., Ren, X., et al. (2015). Distribution of 8 periodontal microorganisms in family members of Chinese patients with aggressive periodontitis. *Arch. Oral Biol.* 60(3)**,** 400-407. doi: 10.1016/j.archoralbio.2014.11.015.

He, J.-y., Qi, G.-g., Huang, W.-j., Sun, X.-d., Tong, Y., Peng, C.-m., et al. (2013). Short-term microbiological effects of scaling and root planing and essential-oils mouthwash in Chinese adults. *J Zhejiang Univ Sci B* 14(5)**,** 416-425. doi: 10.1631/jzus.B1200350.

Huang, Z., Ma, X., Chen, S., Huang, F., and Huang, Z. (2014). Analysis of the diversity of oral bacteria in young adults with chronic periodontitis. *Journal of Pure and Applied Microbiology* 8(2)**,** 1453-1459.

Kageyama, S., Takeshita, T., Asakawa, M., Shibata, Y., Takeuchi, K., Yamanaka, W., et al. (2017). Relative abundance of total subgingival plaque-specific bacteria in salivary microbiota reflects the overall periodontal condition in patients with periodontitis. *PLoS One* 12(4)**,** e0174782. doi: 10.1371/journal.pone.0174782.

Lakio, L., Antinheimo, J., Paju, S., Buhlin, K., Pussinen, P.J., and Alfthan, G. (2009). Tracking of plasma antibodies against Aggregatibacter actinomycetemcomitans and Porphyromonas gingivalis during 15 years. *J. Oral Microbiol.* 1**,** 10.3402/jom.v3401i3400.1979. doi: 10.3402/jom.v1i0.1979.

Li, Y., Tan, X., Zhao, X., Xu, Z., Dai, W., Duan, W., et al. (2020). Composition and function of oral microbiota between gingival squamous cell carcinoma and periodontitis. *Oral Oncol.* 107**,** 104710. doi: 10.1016/j.oraloncology.2020.104710.

Nieminen, A., Sirén, E., Wolf, J., and Asikainen, S. (1995). Prognostic criteria for the efficiency of non-surgical periodontal therapy in advanced periodontitis. *J. Clin. Periodontol.* 22(2)**,** 153-161. doi: 10.1111/j.1600-051X.1995.tb00127.x.

Noguera-Julian, M., Guillén, Y., Peterson, J., Reznik, D., Harris, E.V., Joseph, S.J., et al. (2017). Oral microbiome in HIV-associated periodontitis. *Medicine* 96(12)**,** e5821-e5821. doi: 10.1097/MD.0000000000005821.

O'Brien-Simpson, N.M., Burgess, K., Brammar, G.C., Darby, I.B., and Reynolds, E.C. (2015). Development and evaluation of a saliva-based chair-side diagnostic for the detection of Porphyromonas gingivalis. *J. Oral Microbiol.* 7**,** 29129-29129. doi: 10.3402/jom.v7.29129.

Petit, M.D.A., van Steenbergen, T.J.M., Timmerman, M.F., de Graaff, J., and van der Velden, U. (1994). Prevalence of periodontitis and suspected periodontal pathogens in families of adult periodontitis patients. *J. Clin. Periodontol.* 21(2)**,** 76-85. doi: 10.1111/j.1600-051X.1994.tb00283.x.

Quirynen, M., Mongardini, C., De Soete, M., Pauwels, M., Coucke, W., Van Eldere, J., et al. (2000). The rôle of chlorhexidine in the one-stage full-mouth disinfection treatment of patients with advanced adult periodontitis. *J. Clin. Periodontol.* 27(8)**,** 578-589. doi: 10.1034/j.1600-051x.2000.027008578.x.

Quirynen, M., Mongardini, C., Pauwels, M., Bollen, C.M.L., Van Eldere, J., and Van Steenberghe, D. (1999). One Stage Full- Versus Partial-Mouth Disinfection in the Treatment of Chronic Adult or Generalized Early-Onset Periodontitis. *J. Periodontol.* 70(6)**,** 646-656. doi: 10.1902/jop.1999.70.6.646.

Quirynen, M., and Van Assche, N. (2011). Microbial changes after full-mouth tooth extraction, followed by 2-stage implant placement. *J. Clin. Periodontol.* 38(6)**,** 581-589. doi: 10.1111/j.1600-051X.2011.01728.x.

Saygun, I., Nizam, N., Keskiner, I., Bal, V., Kubar, A., Açıkel, C., et al. (2011). Salivary infectious agents and periodontal disease status. *J. Periodontal Res.* 46(2)**,** 235-239. doi: 10.1111/j.1600-0765.2010.01335.x.

Timmerman, M.F., Van der Weijden, G.A., Abbas, F., Arief, E.M., Armand, S., Winkel, E.G., et al. (2000). Untreated periodontal disease in Indonesian adolescents. *J. Clin. Periodontol.* 27(12)**,** 932-942. doi: 10.1034/j.1600-051x.2000.027012932.x.

Timmerman, M.F., Van der Weijden, G.A., Armand, S., Abbas, F., Winkel, E.G., Van Winkelhoff, A.J., et al. (1998). Untreated periodontal disease in Indonesian adolescents. *J. Clin. Periodontol.* 25(3)**,** 215-224. doi: 10.1111/j.1600-051X.1998.tb02431.x.

van Steenbergen, T.J.M., Petit, M.D.A., Scholte, L.H.M., van der Velden, U., and de Graaff, J. (1993). Transmission of Porphyromonas gingivalis between spouses. *J. Clin. Periodontol.* 20(5)**,** 340-345. doi: 10.1111/j.1600-051X.1993.tb00370.x.

Van Winkelhoff, A.J., Van der Velden, U., Clement, M., and De Graaff, J. (1988). Intra-oral distribution of black-pigmented Bacteroides species in periodontitis patients. *Oral Microbiol. Immunol.* 3(2)**,** 83-85. doi: 10.1111/j.1399-302X.1988.tb00087.x.

von Troil-Lindén, B., Saarela, M., Mättö, J., Alaluusua, S., Jousimies-Somer, H., and Asikainen, S. (1996). Source of suspected periodontal pathogens re-emerging after periodontal treatment. *J. Clin. Periodontol.* 23(6)**,** 601-607. doi: 10.1111/j.1600-051X.1996.tb01831.x.

Von Troil-Lindén, B., Torkko, H., Alaluusua, S., Wolf, J., Jousimies-Somer, H., and Asikainen, S. (1995). Periodontal findings in spouses. *J. Clin. Periodontol.* 22(2)**,** 93-99. doi: 10.1111/j.1600-051X.1995.tb00119.x.

Xu, Y., Selerio-Poely, T., and Ye, X. (2018). Clinical and microbiological effects of egg yolk antibody against Porphyromonas gingivalis as an adjunct in the treatment of moderate to severe chronic periodontitis: a randomized placebo-controlled clinical trial. *J Periodontal Implant Sci* 48(1)**,** 47-59. doi: 10.5051/jpis.2018.48.1.47.

Zambon, J.J., Reynolds, H.S., and Slots, J. (1981). Black-pigmented Bacteroides spp. in the human oral cavity. *Infect. Immun.* 32(1)**,** 198-203. doi: 10.1128/IAI.32.1.198-203.1981.

Zhang, W., Wang, W., Chu, C., Jing, J., Yao, N.A., Sun, Q., et al. (2021). Clinical, inflammatory and microbiological outcomes of full-mouth scaling with adjunctive glycine powder air-polishing: A randomized trial. *J. Clin. Periodontol.* 48(3)**,** 389-399. doi: 10.1111/jcpe.13400.
